# Validation of a new classifier for the automated analysis of the human epidermal growth factor receptor 2 (HER2) gene amplification in breast cancer specimens

**DOI:** 10.1186/1746-1596-8-17

**Published:** 2013-02-04

**Authors:** Daniela Furrer, Simon Jacob, Chantal Caron, François Sanschagrin, Louise Provencher, Caroline Diorio

**Affiliations:** 1 Unité de recherche en santé des populations, Centre de recherche du CHU de Québec, Hôpital du St-Sacrement, 1050 chemin Ste-Foy, Quebec City, QC, G1S 4L8, Canada; 2 Service de Pathologie, Hôpital du St-Sacrement, 1050 chemin Ste-Foy, Quebec City, QC, G1S 4L8, Canada; 3 Centre des Maladie du Sein Deschênes-Fabia, Hôpital du St-Sacrement, 1050 chemin Ste-Foy, Quebec City, QC, G1S 4L8, Canada; 4 Département de biologie moléculaire, de biochimie médicale et de pathologie, Faculté de Médecine, Université Laval, 1050 avenue de la Médecine, Quebec City, QC, G1V 0A6, Canada; 5 Département de chirurgie, Faculté de Médecine, Université Laval, 1050 avenue de la Médecine, Quebec City, QC, G1V 0A6, Canada; 6 Département de médecine sociale et préventive, Faculté de Médecine, Université Laval, 1050 avenue de la Médecine, Quebec City, QC, G1V 0A6, Canada

**Keywords:** Fluorescence *in situ* hybridization (FISH), Trastuzumab, HER2, Image analysis, Tile-sampling analysis, Nuclei-sampling analysis, Nuclei analysis, Accuracy, Breast cancer

## Abstract

Amplification of the human epidermal growth factor receptor 2 (HER2) is a prognostic marker for poor clinical outcome and a predictive marker for therapeutic response to targeted therapies in breast cancer patients. With the introduction of anti-HER2 therapies, accurate assessment of HER2 status has become essential. Fluorescence *in situ* hybridization (FISH) is a widely used technique for the determination of HER2 status in breast cancer. However, the manual signal enumeration is time-consuming. Therefore, several companies like MetaSystem have developed automated image analysis software. Some of these signal enumeration software employ the so called “tile-sampling classifier”, a programming algorithm through which the software quantifies fluorescent signals in images on the basis of square tiles of fixed dimensions. Considering that the size of tile does not always correspond to the size of a single tumor cell nucleus, some users argue that this analysis method might not completely reflect the biology of cells. For that reason, MetaSystems has developed a new classifier which is able to recognize nuclei within tissue sections in order to determine the HER2 amplification status on nuclei basis. We call this new programming algorithm “nuclei-sampling classifier”. In this study, we evaluated the accuracy of the “nuclei-sampling classifier” in determining HER2 gene amplification by FISH in nuclei of breast cancer cells. To this aim, we randomly selected from our cohort 64 breast cancer specimens (32 nonamplified and 32 amplified) and we compared results obtained through manual scoring and through this new classifier. The new classifier automatically recognized individual nuclei. The automated analysis was followed by an optional human correction, during which the user interacted with the software in order to improve the selection of cell nuclei automatically selected. Overall concordance between manual scoring and automated nuclei-sampling analysis was 98.4% (100% for nonamplified cases and 96.9% for amplified cases). However, after human correction, concordance between the two methods was 100%. We conclude that the nuclei-based classifier is a new available tool for automated quantitative HER2 FISH signals analysis in nuclei in breast cancer specimen and it can be used for clinical purposes.

## Introduction

The human epidermal growth factor receptor 2 (HER2) gene is located on chromosome 17 and encodes a transmembrane tyrosine kinase receptor protein [1]. HER2 gene amplification and receptor overexpression, which occur in 15% to 20% of human breast cancers, are important prognostic markers for poor prognosis, including a more aggressive disease and a shorter survival [2]. Moreover, HER2-positive status is a predictive marker of response to trastuzumab therapy in both metastatic and adjuvant settings [3,4]. An accurate evaluation of HER2 status is therefore crucial for identification of patients who would most likely benefit from targeted anti-HER2 therapies. Currently, there are several Food and Drug Administration (FDA)-approved methods to evaluate HER2 status, such as immunohistochemical (IHC) assessment of HER2 protein expression or evaluation of HER2 gene amplification using *in situ* hybridization (ISH), most commonly, fluorescent ISH (FISH) [5,6]. FISH assay is considered to be one of the reference methods for HER2 evaluation in breast cancer, as it accurately predicts response to trastuzumab therapy [7]. Patients are eligible for trastuzumab therapy when their breast cancer specimens are positive at IHC (i.e. 3+) and/or amplified at FISH (ratio > 2.2). However, patients whose tumor specimen is equivocal at FISH (ratio between 1.8 and 2.2) but whose ratio is ≥ 2.0 represent also potential candidates for targeted treatment.

The classical evaluation method for gene amplification, the manual signal enumeration by visual estimation, is a rather time-consuming analysis. Therefore, several companies have developed automated signal quantification systems, which operate through a computer with scanning and image analysis software like Metafer 4 produced by MetaSystems [8,9]. The programming algorithm (the so called “classifier”) currently used by the latter determines the ratio between the average copy number for HER2 to average copy number for chromosome 17 (CEP17) on the basis of equi-sized square tiles [10]. This programming algorithm is defined as “tile-sampling classifier”. However, as the size of tile does not always correspond to the size of a single tumor cell nucleus, some postulate that results obtained might therefore not completely reflect the biology of single cells. To encounter this statement, MetaSystems have recently developed a new programming algorithm, the “nuclei-sampling classifier”, which is able to automatically quantify fluorescent signals in nuclei within tissue sections. In this study, we have compared results obtained with the reference method, the manual scoring, with those obtained with the new nuclei-sampling classifier from MetaSystems in 64 clearly nonamplified (n = 32) and clearly amplified (n = 32) breast cancer specimens.

## Material and methods

### Case selection

A total of 4641 invasive breast cancer cases were identified among all specimens that were examined by FISH between 2009 and 2012 at the Service de Pathologie at CHU-Hôpital du Saint-Sacrement, Québec, Canada. Of these examined cases, 3802 were nonamplified, 636 were amplified and 203 were equivocal. After Ethical Review Board approval, we randomly selected 32 clearly nonamplified and 32 clearly amplified cases among all nonamplified (n = 3802) and amplified (n = 636) cases from our cohort and 32 cases among all equivocal cases (n = 203).

### Fluorescence *in situ* hybridization

HER2 gene copy number was evaluated using the FDA-approved PathVysion™ HER2 DNA Probe kit (Abbott Molecular, Des Plaines, IL/ Inter Medico, Markham, Canada), according to manufacturer’s directions. Briefly, deparaffinized 4-μm sections were immersed in 0.2 N HCl for 20 minutes. Slides were placed in pre-treatment solution at 98°C for 30 minutes and then subjected to protease digestion at 37°C for 5 minutes. Slides were hybridized with PathVysion HER2 DNA probe mixture containing a HER2 DNA probe (labeled with Spectrum Orange) and a CEP17 DNA probe (labeled with Spectrum Green). The CEP17 DNA probe allows for a correction of HER2 gene copy number to the number of copies of chromosome 17. Slide glass coverslips were applied and sealed with rubber cement. Slides were then denatured at 74°C for 2 minutes and hybridized overnight at 37°C in a humidified hybridization chamber (ThermoBrite™, Abbott Molecular/ DAKO, Glostrup, Denmark). On the following day, slides were washed in a post-hybridization buffer at 73.5°C for 2 minutes and dried in the dark. Nuclei were subsequently counterstained with 10 μL of 4’,6-diamidino-2-phenylindole (DAPI). Slides were stored in the dark at 4°C until signal enumeration.

### Manual scoring (reference method)

Analysis of fluorescent signals were performed with an epifluorescence microscope Axio Imager M1 (Zeiss, Göttingen, Germany), equipped with a triple-band filter (DAPI/green/orange). Slides were visually scored according to the protocol described in PathVysion HER2 package insert. Briefly, slides were first analyzed at low magnification using a DAPI filter to identify areas of invasive carcinoma showing optimal tissue digestion and non-overlapping nuclei within tumor areas selected by a pathologist. Slides were analyzed by trained technologists, and results were validated by two breast pathologists with experience in FISH interpretation (SJ or CC). Results were reported according to the ASCO/CAP and Canadian HER2 scoring guidelines [5,6]. Specimens with a HER2/CEP17 ratio of > 2.2 were considered amplified, those with a ratio between 1.8 and 2.2 were considered equivocal, while specimens with a ratio of <1.8 were considered nonamplified [5,6]. Average copy numbers of HER2 and CEP17 from at least 20 randomly selected nuclei, each with at least one HER2 and one CEP17 signal, from different areas of the invasive carcinoma were counted and HER2/CEP17 ratio calculated. For equivocal cases, ratios were calculated from at least 60 tumor cells. Equivocal cases were counted by two independent trained technologists and reviewed by the pathologists of this study.

### Tile-sampling classifier

**Figure 1:**
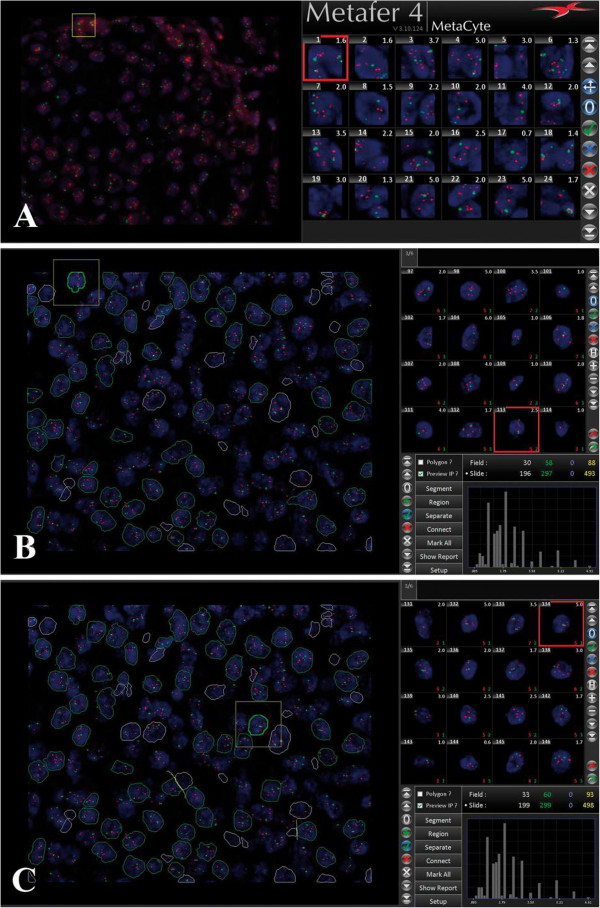
**Image analysis of fluorescence signals. A**). Automated quantitative image analysis through the tile-sampling classifier. Non-overlapping square tile were placed on DAPI-counterstain image in order to maximize the nuclear material covered. Each tile may contain a single nucleus or portions of one or more nuclei. The software effectuated the spot count in each tile. **B**). Quantitative image analysis through the automated nuclei-sampling classifier. The classifier automatically recognized individual nuclei in counterstain image (same image as in Figure 1A). Nuclei that were automatically recognized and considered in the analysis showed green outlines, while nuclei that were automatically recognized but not considered in the analysis had a white outline. The software effectuated spot count in each nucleus. **C**). After completion of the automated image analysis, the user could improve the selection of automatically selected nuclei via interaction with the software. In this particular case, some nuclei that were not yet considered during the automatic segmentation (Figure 1B) were selected by the user, some automatically selected nuclei were deleted and some overlapping nuclei were divided (yellow line between two overlapping nuclei).

Automated fluorescence signal analysis was performed through the FDA-approved MetaSystems™ image analysis system. The capture station is composed of a scanner, an automated fluorescence microscope, a M4+ CCD camera with Mercury Lamp HBO 100 and a computer with the microscope scanning and analysis software Metafer 4 with “tile-sampling” method (MetaSystems, Altussheim, Germany). The Local Area Network (LAN)-connected capture station worked on PC Intel Core2 Duo CPU E8500 3.16 GHz , 3.25 GB of RAM server, with 1280x1024 pixel size. After selection of 5 to 10 non-overlapping fields of infiltrating carcinoma by trained technologists within tumor areas identified by a pathologist, fields’ images were automatically captured and analyzed by the software. In analogy to manual scoring, representative images with optimal tissue digestion and non-overlapping nuclei were selected. All images were captured at 400x microscope magnification. Minimal integration time was 0.04 seconds. Image size was 1088 × 880 pixels × 8 bits (between 40 and 90 MB, in MetaSystems format. TRN, with 255 gray levels). A classifier is a programming algorithm which defines how images are captured and analyzed by the software. The classifier used for this type of analysis, the “tile-sampling classifier”, permitted extensive tumor sampling by placing non-overlapping equi-sized square tiles in counterstain images (DAPI image). Square tiles were 71 pixels in size and were generally on the order of the size of one or two nuclei. Classifier placed tiles in regions of images where nuclear material was the highest, in order to include as much nuclear material and as little empty space as possible in tiles. Classifier recognized these regions through the DAPI filter. Aim of this strategy was to maximize the total fluorescence intensity covered (Figure 1A) [10]. Captured images underwent image processing through several filters (Gaussian smoothing filter, TopHat filter and Laplace) and application of a counterstain mask [10]. Image analysis software carried out FISH spot counting through analysis of fluorescence signals present within tiles using several signal colour channels. HER2 spots were recognized through Spectrum Orange filter and were defined as object with an area of 0.05 μm^2^, a distance of 0.8 μm between objects and an intensity of 33% after image processing. CEP17 spots were recognized through Spectrum Green filter and were defined as object with an area of 0.18 μm^2^, a distance of 0.5 μm between objects and an intensity of 30% after image processing. Single tiles were rejected by the classifier when less than 40% of the surface was occupied with nuclei, or when they contained only one fluorescent signal (only one orange or only one green fluorescent signal). Cases were rejected when they contained less than 32 tiles. Time consumption for analysis by tile-sampling classifier on a local station was between 3 and 5 minutes. Image analysis software calculated signal ratio by dividing the average HER2 and CEP17 spot number per tile [10]. Results were reported using the same scoring guidelines as for manual scoring (nonamplified, equivocal, amplified).

### Nuclei-sampling classifier

The same images produced during the tile-sampling analysis were analyzed with a new Metafer 4 classifier (MetaSystems). The new classifier was tested on the analysis station, which was composed of a PC Intel Core2 Duo CPU E4600 2.4 GHz, 1 GB of RAM server with 1280x1024 pixel size. Analysis station was LAN-connected with capture station. Size of images was identical as for the tile-sampling analysis, as we used the same images (1088 × 880 pixels × 8 bits). This new classifier automatically recognized individual cells in counterstain image through segmentation of nuclei, i.e. the outlining of individual nucleus. Nuclei were recognized by the classifier when they had an object area between 12 μm^2^ and 400 μm^2^ with certain roundness in DAPI images. Color of nuclei outlines informed the user of which nuclei have been considered for analysis. Nuclei that were automatically segmented and considered in analysis showed green outlines, while pre-segmented nuclei (nuclei that were automatically segmented but not considered in the analysis) were represented by a white outline (Figure 1B). The software recognized appropriate nuclei for analysis on the basis of size and shape of nuclei and on quality of fluorescent signals. In this paper, we defined this method as the “nuclei-sampling analysis” to differentiate it from the “tile-sampling analysis”. The nuclei-sampling analysis was performed blinded from results obtained by manual scoring and tile-sampling analysis.

This automated nucleus segmentation was followed by an optional human correction, in which the user interacted with interface in order to improve selection of cell nuclei automatically selected by the software. This optional interactive phase required an interactive touch screen (e.g. the WACOM® DTU-2231, 1920 × 1080 pixels, MetaSystems). The interaction was performed using a mouse or an interactive pen display. The user utilized the following interaction options: addition of nuclei (not yet considered during the automated segmentation) to be analyzed, selection of pre-segmented nuclei (nuclei that were recognised by the software but not considered in the analysis), deletion of automatically selected nuclei, partition of overlapping nuclei, or connection of separated objects (for instance parts of the same nucleus that were accidentally separated during the automated segmentation) (Figure 1C). Any of these operations led to automatic updates of signal ratio results. The software carried out FISH spot counting for both the automated and the human corrected nuclei-sampling analyses in the same way as for the tile-sampling classifier. Again, results for both automated and human corrected nuclei-sampling analyses were reported using the same scoring guidelines as for manual scoring (nonamplified, equivocal, amplified).

## Results

### Validation of the nuclei-sampling classifier in nonamplified and amplified breast cancer specimens

In order to validate the new classifier, we examined the concordance of results obtained by manual scoring and by nuclei-sampling analysis in 32 clearly nonamplified and 32 clearly amplified cases, chosen randomly in our cohort of breast cancer patients. Our selection criteria fulfill the requirements of the ASCO/CAP and Canadian recommendations for HER2 testing in breast cancer for the validation of a new test. Indeed, these guidelines recommend that a new test has to be compared with a reference test in at least 25 samples, ideally by using 50% cases that are clearly positive and 50% cases that are clearly negative [5,6]. Table 1 shows the comparison between results obtained by manual scoring, the reference method, and the tile-sampling analysis and nuclei-sampling analysis in these 64 breast cancer specimens. For the nuclei-sampling analysis, concordance between reference method (manual scoring) and results obtained with the automated analysis (before human correction) was 100% for nonamplified cases and 96.9% for amplified cases. Overall concordance rate was 98.4%. One case out of 32 considered as amplified by manual scoring was considered as borderline equivocal (ratio of 2.2) with the nuclei-sampling analysis. However, after human correction, concordance between manual scoring and nuclei-sampling analysis was 100% for both the nonamplified and the amplified cases. Concordance between manual scoring and tile-sampling analysis was 100% for both the nonamplified and the amplified cases.

**Table 1:** **Comparison of results obtained by different methods for nonamplified and amplified cases** (**n** = **64**)

**Manual scoring**	**Tile**-**sampling analysis**			**Nuclei**-**sampling analysis**					
				**Automated analysis**			**After human correction**		
	**Nonamplified**	**Equivocal**	**Amplified**	**Nonamplified**	**Equivocal**	**Amplified**	**Nonamplified**	**Equivocal**	**Amplified**
Nonamplified	32	0	0	32	0	0	32	0	0
Amplified	0	0	32	0	1	31	0	0	32

### Determination of the accuracy of the nuclei-sampling classifier on special specimens

**Figure 2:**
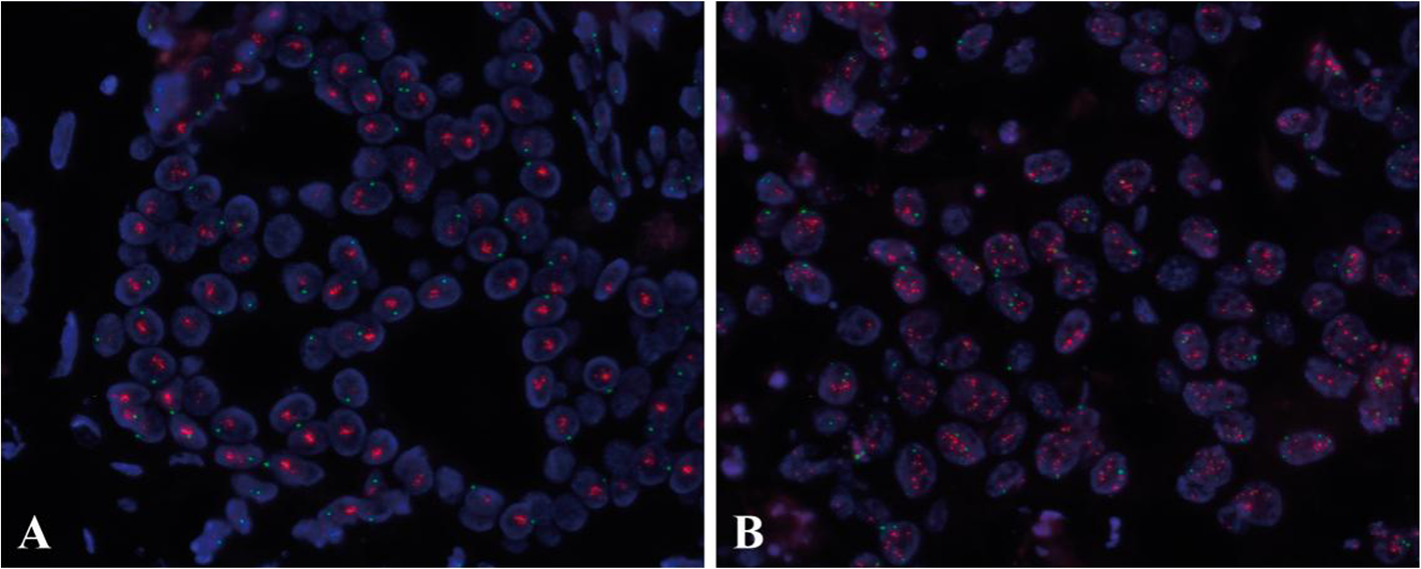
**HER2 fluorescence *in situ *hybridization in amplified cases. A**). Amplified case showing Homogeneously Staining Regions (HSR). HSR are large clusters of HER2 fluorescence signals indicating the presence of HER2 gene amplification in tandem repeats. **B**). Amplified case without HSR. HER2 signals are identifiable as single spots.

In a closer analysis of the randomly selected amplified cases, we observed that all examined cases were amplified with Homogeneously Staining Regions (HSR). HSR are large clusters of HER2 fluorescence signals indicating the presence of HER2 gene amplification in tandem repeats (Figure 2A). In samples in which HSR occurred and individual signals could not be counted, the software adopted a different spot counting analysis, which evaluated the signal area in the HER2 channel instead of individual spot counts [10]. As in the population of this study approximately 4% of amplified cases do not show HSR (Figure 2B), we examined the accuracy of the new classifier also on these less common cases. In our cohort, we identified 28 amplified cases without HSR. Although cases were recognized as amplified without HSR on the basis of results obtained through tile-sampling analysis, manual scoring represented the reference method also for these cases. Results for amplified cases without HSR are summarized in Table 2. Of these 28 amplified cases without HSR, 21 cases were classified as amplified with the automated nuclei-sampling analysis, and this number increased to 24 after human correction. Concordance between manual scoring and nuclei-sampling analysis was 75% (21/28). Of the 7 discordant cases that showed a ratio ≤ 2.2 with the automated nuclei-sampling analysis, 4 had a ratio between 2.0 and 2.2, whereas 3 had a ratio smaller than 2.0. After human correction, the concordance between the manual scoring and the nuclei-sampling analysis was 86% (24/28). Of the 4 discordant cases that showed a ratio ≤ 2.2 with nuclei-sampling analysis after human correction, 3 cases showed a ratio between 2.0 and 2.2, whereas 1 case showed a ratio smaller than 2.0. Concordance between tile-sampling analysis and manual scoring method was 100%. This result is not surprising, as the samples without HSR were identified on the basis of results obtained through tile-sampling analysis.

**Table 2:** **Comparison of results obtained by different methods for amplified cases without HSR** (**n** = **28**)

**Manual scoring**	**Tile**-**sampling analysis**			**Nuclei**-**sampling analysis**					
				**Automated analysis**			**After human correction**		
	<**2**.**0**	**2**.**0** – **2**.**2**	>**2**.**2**	<**2**.**0**	**2**.**0** – **2**.**2**	>**2**.**2**	<**2**.**0**	**2**.**0** – **2**.**2**	>**2**.**2**
> 2.2	0	0	28	3	4	21	1	3	24

*HSR* = Homogeneously Staining Regions.

### Determination of the accuracy of the nuclei-sampling classifier on equivocal specimens

To further evaluate the accuracy of the new classifier, we examined 32 equivocal cases. However, only 29 cases have been analyzed, since in 3 cases, images were of very poor quality. Equivocal cases constitute approximately 5% of our cohort. Results for equivocal cases are summarized in Table 3. Of these 29 equivocal cases, 9 cases were classified as equivocal, 19 as nonamplified and one case was classified as amplified with tile-sampling analysis. With nuclei-sampling analysis after human correction, 17 cases were classified as equivocal, 11 as nonamplified and one case was classified as amplified. Concordance between manual scoring and tile-sampling analysis was 31% (9/29), whereas the concordance between manual scoring and nuclei-sampling analysis after human correction was 59% (17/29). Among the 15 cases whose ratio was ≥ 2.0 at the manual scoring, 3 (20%) were also ≥ 2.0 at the tile-sampling method, whereas 6 out of these 15 cases (40%) were ≥ 2.0 at the nuclei-sampling method after human correction. Among the 14 cases whose ratio was < 2.0 at the manual scoring, 13 (93%) were also < 2.0 at the tile-sampling method, whereas 12 out of these 14 cases (86%) were < 2.0 at the nuclei-sampling method after human correction.

**Table 3:** **Comparison of results obtained by different methods for equivocal cases** (**n** = **29**)

**Manual scoring**	**Tile**-**sampling analysis**				**Nuclei**-**sampling analysis**, **after human correction**			
	<**1**.**8**	≥ **1**.**8** – < **2**.**0**	**2**.**0** – **2**.**2**	>**2**.**2**	<**1**.**8**	≥ **1**.**8** – < **2**.**0**	**2**.**0** – **2**.**2**	>**2**.**2**
≥ 1.8 - < 2.0	12	1	1	0	7	5	1	1
2.0 – 2.2	7	5	2	1	4	5	6	0

### Reproducibility of results

All cases analyzed in this study (clearly nonamplified, clearly amplified, equivocal and amplified cases without HSR) were assessed blindly by a second independent observer. The results obtained by the two observers were similar (data not shown).

## Discussion

Our results showed an excellent concordance between manual scoring, our reference method, and nuclei-sampling analysis for clearly nonamplified and clearly amplified cases. Indeed, the concordance of results for nonamplified cases was 100%, both for the automated and the human corrected nuclei-sampling analyses. For amplified cases, the concordance between the two methods was 96.9% for the automated nuclei-sampling analysis and rose to 100% following human correction. These concordance rates with manual scoring results fulfill the ASCO/CAP requirements of concordance greater than 95% for clearly amplified and nonamplified cases [6].

Our results are consistent to those obtained by Theodosiou and collaborators [11]. In their study, they examined the utility of an image analysis software (EIKONA3D, Alpha Tec Ltd) for the evaluation of HER2 amplification in nuclei in 100 breast cancer cases from two institutions. Similar to the analysis software presented here, the user had the possibility to manually correct the results obtained through the automated nuclei segmentation. They found a very good overall concordance (92.8%) between the results obtained by manual scoring by an expert and those obtained with the image analysis software. Similar to our results, the concordance for nonamplified cases was 100%, whereas the concordance for amplified cases was lower, 74.1% [11].

In this work, we validated the new Metafer 4 classifier in 64 breast cancer specimens (32 nonamplified and 32 amplified cases), chosen randomly among eligible clearly nonamplified and amplified cases of our cohort, as required from the ASCO/CAP and the Canadian guidelines for HER2 testing in breast cancer for validation of a new test. Accordingly to these recommendations, a new test has to be compared with the reference test in at least 25 samples, ideally by using 50% cases unequivocally positive and 50% cases unequivocally negative [5,6]. The new classifier evaluated here was able to recognize cell nuclei on the image and therefore to calculate HER2 FISH ratio on nucleus basis. Moreover, this new classifier allowed the user to interact with the software during an optional interactive phase, in order to improve the selection of cells automatically selected by the software. In this study, we defined this method as “nuclei-sampling analysis” to differentiate it from “tile-sampling analysis”, which was performed with the Metafer 4 classifier currently used. This classifier, in fact, calculated HER2 FISH ratio on the basis of equi-sized tiles placed by the software on images. In order to validate this new classifier, we compared results obtained through manual scoring of slides, considered as the reference method, with those obtained through nuclei-sampling analysis. Moreover, we analyzed the accuracy of both the automated and the human corrected nuclei-sampling analyses.

As all randomly selected amplified cases analyzed in this study were amplified with HSR, we decided to evaluate the accuracy of this new classifier on the less common amplified cases without HSR. These cases represented indeed about 4% of all amplified cases in our cohort of breast cancer patients. For the 28 cases without HSR that we have analyzed, concordance between manual scoring and automated nuclei-based method was 75%. After human correction, concordance between the two methods rose to 86%. Considering that patients whose specimen is equivocal at FISH (ratio between 1.8 and 2.2) but whose ratio is ≥ 2.0 represent also potential candidates for trastuzumab treatment, 4 patients out of 7 discordant cases at the automated nuclei-sampling analysis, and 3 cases out of 4 discordant cases at the nuclei-sampling analysis after human correction would therefore be eligible to receive a targeted treatment. We noticed that some discordant cases were polysomic or monosomic (4 out of 7 discordant cases) and we postulate that this aneuploidy status could explain the discordance. It has been reported that biological variance reduces sampling efficiency [12]. Indeed, higher biological variance associated with aneuploidy status could have had an impact on the spot counting by the software and this could explain the discrepancy with results obtained by manual scoring. Moreover, quality of the images of some discordant cases (2 out of 7 discordant cases) was poor (cell nuclei were blurred in the image), which could also be an additional explanation for this discrepancy.

With the aim to further analyse the accuracy of the new classifier, we also examined equivocal cases, which represent about 5% of our cohort population. Overall concordance between manual scoring and tile-sampling method was 31%, whereas concordance between manual scoring and nuclei-sampling method after human correction was 59%. If equivocal cases were splitted in those with ratio ≥ 2.0 and those with ratio < 2.0, we noticed that twice as many cases were correctly classified with a ratio ≥ 2.0 using nuclei-sampling method after human correction as compared to tile-sampling method. So even if concordance between manual scoring and nuclei-sampling method was not optimal, these results suggest that nuclei-sampling method is more reliable than tile-sampling method for the identification of patients who could potentially benefit from targeted anti-HER2 therapies. Similar to the amplified cases without HSR, we also noticed that some discordant cases were aneuploid (4 out of 12 discordant cases). Also, in 2 out of 12 discordant cases the quality of images was poor (cell nuclei were blurred in images).

Tile-sampling method has been developed by MetaSystems and other companies in order to overcome the difficulties that are frequently encountered when fluorescent signals are enumerated via automated image analysis software. Firstly, reliable separation of overlapping nuclei in tissue sections is very difficult, especially in dense packed tissues like breast cancer. Secondly, it is arduous for image analysis software to automatically distinguish distinct cell populations (normal and tumor cells) present in analyzed fields. To overcome these difficulties, the Metafer 4 software places non-overlapping tiles of equal size on images in order to cover the majority of nuclear material and therefore quantify fluorescent signals. Moreover, a ratio estimation algorithm was introduced with the aim to improve the accuracy of results of the automated analysis in samples in which distinct cell populations are present [10,13].

Although the tile-sampling analysis is in general well performing [8,9], the nuclei-based analysis offers some advantages compared to the tile-based analysis. Firstly, the way in which the new classifier selects nuclei for analysis coincides better to what the user does when the user is analyzing a sample. Whereas the nuclei-sampling analysis recognizes cell nuclei, the tile-sampling analysis places equi-sized tiles on the image. In addition, as the size of tile does not always correspond to the size of a single nucleus, nuclei are often truncated during tile-sampling. As a consequence, one single tile may contain signals from multiple nuclei or only part of a nucleus. This can be disadvantageous especially in cases of chromosome 17 monosomy or polysomy, where exact number of CEP17 signal per cell is relevant. Secondly, the nuclei-based analysis offers the advantage that the user can improve the selection of cell nuclei that have been automatically selected by the software through active interaction with the software. During the interactive phase, the user can add nuclei that were not considered during the automated selection, delete unsuitable nuclei, divide overlapping nuclei or connect separated pieces of the same nucleus. This optional, interactive phase requires additional time, in average 7 minutes for equivocal cases and amplified cases without HSR and 4 minutes for nonamplified cases and amplified cases with HSR, but it is very helpful and effective especially in difficult cases, for instance in samples with abundant stroma or intermixed normal cells. In fact, we observed a better concordance between results obtained by the reference method and those obtained with the nuclei-based analysis after the interactive phase, compared to results obtained with the automated analysis. Theodosiou and collaborators observed similar results using a similar method. In their hand, manual correction required up to 5 minutes for each case (nonamplified and amplified cases) and it was particularly useful in cases with low image quality [11]. We noticed that among all functions that the user could choose during the interactive phase, the delete function was the most effective one. In fact, when discordant cases were evaluated blindly by a second independent observer who used exclusively the delete function, results obtained by the two observers were similar (data not shown). We may therefore conclude that the delete function is very effective in improving the results obtained with the automated nuclei-based analysis. Moreover, the time necessary for human correction can additionally be reduced if only the delete function is used during the interactive phase (6 minutes in average for equivocal cases and amplified cases without HSR). The automated nuclei-sampling analysis required between 3 and 5 minutes per case, depending on the cellularity of images. Our image analysis software is slower compared to others, for example Matlab, which required 3.5 seconds for analysis of a single image on local server [14]. As we analyzed between 5 and 10 images for each case, Matlab would have taken between 17.5 and 35 seconds to evaluate a case.

In this study, the reference method was represented by the manual scoring of specimen. A closer examination of nuclei automatically selected by the software during nuclei-based analysis allowed us to observe how the user can also be biased when the user is analysing a case. In particular, human eyes have a tendency to pay more attention to those cell nuclei in which more fluorescent signals are present. One could therefore argue that human brain considers those nuclei more attractive and preferentially chooses them during the manual signal enumeration. Nuclei-based analysis, on the contrary, selects nuclei on the basis of the shape of cell nuclei and on quality of fluorescent signals and is therefore more “neutral” in the choice of the nuclei. Therefore, eligible nuclei that have less fluorescent signals (and may be judged as less attractive by human brain) are also taken into account for analysis from software. Opinions on this topic are divergent. Whereas some underline that software do not always select the most appropriate nuclei for analysis [11], others claim that results obtained with automated analysis are more accurate especially in amplified and borderline cases, as manual analysis of HER2 signals can only be estimated when probe signals cluster closely together [15]. Another advantage of image analysis system over manual scoring is that storing of captured images allows archiving of cases for future study.

Some limitations are associated with the new classifier. Accuracy of the new classifier to recognize nuclei is markedly reduced in images with dense packing of cells or in images in which DAPI counterstain is blurred. As mentioned above, results obtained with any quantitative image analysis software depend tremendously on the fields chosen by the observer for analysis. If the fields chosen are not representative of the sample, results obtained by quantitative image analysis can be rather different from those obtained through manual scoring. This issue is common to all diagnostic algorithms. Reliable sampling procedure is prerequisite for diagnostic accuracy in virtual microscopy [12,16].

Standardization of images capture is a central point in the development of a diagnostic algorithm in virtual microscopy [17]. In our study, optimal specification for the capture of images from FISH HER2 slides hybridized with PathVysion™ HER2 DNA Probe kit (image size, size of tiles, identification criteria for HER2 and CEP17 spots, segmentation criteria for nuclei, filtering) has been previously established using over 400 slides (personal communication, Ulrich Klingbeil, MetaSystems). Quality of captured images is in general excellent, since quality and intensity of fluorescence signals are reproducible and background is very low. Dissimilar to other algorithms used in object-related diagnosis [16], fluorescent spot identification is less problematic. In contrary to other structures within tissue that are difficult to be recognized, fluorescent spots are easily identified by the classifier, as they are mostly of the same size and intensity, except for HSR cases. However, spot identification is also reliable in amplified cases with HSR (where spot dimensions can be more variable), since the software adopted a different spot-counting analysis (evaluation of the signal area in the HER2 channel instead of individual spot counts) [10].

Tissue-based diagnosis has been subjected to remarkable changes following the introduction of new technologies. For instance, technological advances in tissue-based diagnosis allow the implementation of digitized images into routine clinical pathology. Virtual pathology has several advantages compared to conventional microscopy. For example, virtual pathology allows archiving of virtual images, promotes continuing education as well as interactive remote consultation between pathologists [18]. Moreover, it has been reported that analysis of digitized slides gives results as accurate as that obtained through conventional microscopy [19,20]. However, one critical point is whether the diagnostic information contained in the virtual slides reliably reflect the real whole slide. In this context, the adopted sampling procedure plays a central role [12]. This is an important point to consider, when the efficacy of virtual diagnostic algorithms are compared [12]. Both the tile-sampling classifier and the nuclei-sampling classifier are based on a stratified and passive sampling method as defined in Kayser et al. [12]. However, whereas the tile-sampling classifier recognizes nuclear material through the DAPI filter (and put square tiles on the image, where the DAPI coloration is the strongest), the nuclei-sampling classifier recognizes single nuclei within tissue on the basis of nuclei characteristics, such as nuclei size and roundness. Spot recognition and spot counting is effectuated in same way for both methods.

In our clinical context, pathologists share virtual images and results via a LAN platform. This form of information sharing represents one of the first steps towards the so called “Grid technology”. A Grid is an open and dynamic communication system consisting of connected nodes (i.e. servers) that are linked together via Internet connections and share certain communication rules in using open standards [21]. The Grid technology will also have an impact on the quality in tissue-based diagnosis as such implementation will require appropriate standardization of legal, medical and technological aspects associated with virtual pathology [17].

## Conclusions

In summary, we observed an excellent concordance between results obtained by manual scoring and those by nuclei-sampling analysis in 32 clearly nonamplified and 32 clearly amplified breast cancer specimens. However, accurate determination of HER2 amplification in equivocal cases (ratio between 1.8 and 2.2) remains a challenge [5,6]. Manual assessment of these cases, therefore, remains the standard procedure. We conclude that the new image analysis software Metafer 4 classifier is a reliable tool to evaluate the unequivocal status of HER2 in breast cancer specimens and it is ready to be implemented in clinics, as it offers several advantages compared to the Metafer 4 classifier currently used. Moreover, although more time-consuming, human correction after the completion of the automated nuclei-sampling analysis is recommended, especially in particular cases (like those with abundant stroma), as this operation leads to an improvement of the results obtained during the automated analysis.

## Abbreviations

HER2: Human epidermal growth factor receptor 2; CEP17: Chromosome 17 centromere; FISH: Fluorescence *in situ* hybridization; ASCO/CAP: American Society of Clinical Oncology/College of American Pathologists; HSR: Homogeneously Staining Regions; FDA: Food and Drug Administration; DAPI: 4′,6-diamidino-2-phenylindole; IHC: Immunohistochemistry.

## Competing interests

The new Metafer 4 software version and the interactive touch screen were kindly provided by MetaSystems.

## Authors’ contributions

DF participated in the conception of the study, data collection, analysis and interpretation of results and wrote the manuscript. SJ, CC, FS and CD participated in the conception of the study, data collection, analysis and interpretation of results and reviewed the manuscript. LP participated in the analysis and interpretation of the results and editing of the manuscript. All authors read and approved the final manuscript.
